# Exo-erythrocytic development of avian malaria and related haemosporidian parasites

**DOI:** 10.1186/s12936-017-1746-7

**Published:** 2017-03-03

**Authors:** Gediminas Valkiūnas, Tatjana A. Iezhova

**Affiliations:** Nature Research Centre, Akademijos 2, LT-08412 Vilnius, Lithuania

**Keywords:** Avian malaria, *Plasmodium*, *Fallisia*, *Haemoproteus*, *Leucocytozoon*, *Akiba*, Exo-erythrocytic development, Pathology

## Abstract

**Background:**

Avian malaria parasites (*Plasmodium* spp.) and related haemosporidians (Haemosporida) are responsible for diseases which can be severe and even lethal in avian hosts. These parasites cause not only blood pathology, but also damage various organs due to extensive exo-erythrocytic development all over the body, which is not the case during *Plasmodium* infections in mammals. However, exo-erythrocytic development (tissue merogony or schizogony) remains the most poorly investigated part of life cycle in all groups of wildlife haemosporidian parasites. In spite of remarkable progress in studies of genetic diversity, ecology and evolutionary biology of avian haemosporidians during the past 20 years, there is not much progress in understanding patterns of exo-erythrocytic development in these parasites. The purpose of this review is to overview the main information on exo-erythrocytic development of avian *Plasmodium* species and related haemosporidian parasites as a baseline for assisting academic and veterinary medicine researchers in morphological identification of these parasites using tissue stages, and to define future research priorities in this field of avian malariology.

**Methods:**

The data were considered from peer-reviewed articles and histological material that was accessed in zoological collections in museums of Australia, Europe and the USA. Articles describing tissue stages of avian haemosporidians were included from 1908 to the present. Histological preparations of various organs infected with the exo-erythrocytic stages of different haemosporidian parasites were examined.

**Results:**

In all, 229 published articles were included in this review. Exo-erythrocytic stages of avian *Plasmodium, Fallisia, Haemoproteus, Leucocytozoon*, and *Akiba* species were analysed, compared and illustrated. Morphological characters of tissue stages that can be used for diagnostic purposes were specified.

**Conclusion:**

Recent molecular studies combined with histological research show that avian haemosporidians are more virulent than formerly believed. The exo-erythrocytic stages can cause severe disease, especially in non-adapted avian hosts, suggesting the existence of a group of underestimated malignant infections. The development of a given haemosporidian strain can be markedly different in different avian hosts, resulting in significantly different virulence. A methodology combining the traditional histology techniques with molecular diagnostic tools is essential to speed research in this field of avian malariology.

## Background

Malaria parasites (species of *Plasmodium*) and related haemosporidians of the order Haemosporida are widespread and diverse pathogens infect many species of the major groups of vertebrates, which are exposed to bites of blood-sucking dipteran insects (Diptera). The latter insects are specific vectors of haemosporidians [1]. Many species of reptiles, birds and mammals are often parasitized, and several species of amphibians and even fish have been reported as hosts of haemosporidians [2–6]. The life cycle of these blood parasites includes the following main obligatory stages: (1) exo-erythrocytic merogony (schizogony); (2) development in blood cells and production of gametocytes, which are infective for vectors; and, (3) sexual process and sporogony occurring in dipteran insects, which inject infective sporozoites and initiate new infections in vertebrate hosts. General descriptions of life cycles of different malaria and related haemosporidian species are available in several books and reviews [2–9]. However, the details of development of these pathogens both in avian hosts and vectors remain unknown for the great majority of the described species. This study reviews knowledge and provides comparative morphological data about exo-erythrocytic development of avian malaria and related haemosporidian parasites of the families Plasmodiidae, Haemoproteidae, Garniidae, and Leucocytozoidae.

Avian malaria parasites and other haemosporidians cause not only blood pathology due to development of high parasitaemia, but also pathology in organs because of the damage caused by exo-erythrocytic stages, which often develop in various non-specialized reticuloendothelial cells and can be found all over the body in susceptible birds, including the brain, eyes, nerves, heart, skeletal muscles, and many others organs and tissues [2–7, 10]. From this point of view, avian malaria caused by some *Plasmodium* species is an even more severe disease than mammalian malaria, during which the exo-erythrocytic development occurs mainly in hepatic cells and usually does not cause disease at this stage of infection [11, 12].

During the last 20 years, numerous studies have addressed the taxonomy, genetic diversity, ecology, evolutionary biology, and genetics of avian haemosporidians (reviewed in [13–19]). The application of sensitive polymerase chain reaction (PCR)-based diagnostic methods, using relatively easy-to-collect samples of infected peripheral blood, provided much new knowledge about these topics, but contributed little information about exo-erythrocytic development. The great majority of recent avian haemosporidian studies only used blood samples to assess haemosporidian infections, which gives no information on the tissue stages. It is important to note that several recent histopathological studies, which combined molecular diagnostic and microscopic research, showed that widespread haemosporidian lineages, particularly of *Haemoproteus* species, may cause severe disease and even mortality in birds due to marked damage by exo-erythrocytic meronts [20–26]. Interestingly, abortive development of tissue stages of haemosporidians in non-adapted avian hosts caused mortality, and that is a new issue related to bird health in studies of exo-erythrocytic development of avian haemosporidians.

Abortive development happens when a parasite invades a ‘wrong’ host, succeeds to develop partially, but cannot complete its full life cycle, resulting in the absence of invasive stages (gametocytes in avian hosts or sporozoites in vectors). The parasite is ‘captured’ by the host during abortive development, which is a dead-end of the infection [27–29]. Infections in ‘wrong’ hosts are usually eliminated rapidly, with minimal or no symptoms, as was reported in non-successful experimental infections [4, 5]. However, if the infection proceeds partially, the immune response to get rid of it may be highly symptomatic. Abortive haemosporidian infections usually are difficult to diagnose because of unusual location and morphology in the ‘wrong’ host, unclear etiology, short-term survival of parasites or rapid mortality of infected hosts [22, 28–30].

In old literature (published before 1995), there are numerous descriptions of lethal avian diseases of unclear etiology caused by tissue stages of haemosporidians [31–43]. The tissue stages in dead birds sometimes resemble the megalomeronts of *Leucocytozoon* species, which is why these diseases were formerly often described as ‘aberrant *Leucocytozoon* infections’ or even ‘*Besnoitia* infections’ [44]. Recent molecular studies and histopathological research indicate that widespread lineages of haemosporidians are responsible for mortality due to the damage caused by tissue stages of these parasites [20–26, 45]. The application of molecular diagnostic tools indicates that haemosporidian infections might kill abnormal vertebrate hosts during exo-erythrocytic development [22, 29]. For example, the prevalence of *Haemoproteus minutus* (Haemoproteidae) infection is high (>50%) in most blackbird *Turdus merula* populations in Europe, and active transmission takes place even in parks of European capitals [26]. The blackbird is adapted to this infection, which is asymptomatic in this host, but *H. minutus* cause lethal disease in captive parakeets in Europe. Abortive tissue stages of this parasite develop in myocardial and skeletal muscles and other organs, but parasitaemia is absent, making it difficult to diagnose such infections by microscopic examination or PCR-based testing solely of blood samples [22, 23]. Presence of haemosporidians can be reported in organs of birds using solely molecular diagnostic tools [46], but this methodology is insufficiently sensitive to understand pathology caused by the parasites. Traditional histological methods remain important in morphological characterization of haemosporidian tissue stages [47]. However, the descriptions and illustrations of these stages are scattered throughout numerous publications, many of which are old, not easy to access and sometimes are difficult to use for comparative parasitology research.

The aims of this review were: (1) to overview main available information on exo-erythrocytic development of avian *Plasmodium* and related haemosporidian parasites; and, (2) to discuss future research priorities in this field of avian malariology. This study provides images of common haemosporidian exo-erythrocytic stages, which can be used during identification of these infections. Information about pathological changes in bird organs is only briefly mentioned; it is available in several reviews [2–5, 48] and its detailed consideration requires separate analysis.

## Methods

### Collection of literature

Mainly, full-length papers published in peer-reviewed journals were considered. Old articles (published before 1995) were collected by the first author during his work in the libraries of Oxford University, Natural History Museum, the Liverpool School of Tropical Medicine, and the International Centre for Avian Haematozoa (now available in the Queensland Museum, Australia) in 1992–1996. Published bibliographies of the avian blood-inhabiting haematozoa [49–51] and reviews on haemosporidian parasites [2–5, 7–9, 52, 53] were also used. Recent peer-reviewed articles have been retrieved from online bibliographic databases PubMed, SCOPUS and Google Scholar using the following keywords: ‘malaria’, ‘birds’, ‘Haemoproteus’, ‘Plasmodium’, ‘Leucocytozoon’, ‘Akiba’, ‘Fallisia’, ‘pathology’, ‘meront’, ‘schizont’. The Boolean operators ‘OR’, ‘AND’, and ‘>’ were used to combine several terms. In all, 229 full text articles and short reports were reviewed. Articles are cited in the review if they explicitly provided data about one of the considered aspects of exo-erythrocytic development or genetic diversity of avian malaria or other haemosporidian parasites; 212 papers containing most representative information about exo-erythrocytic development of these parasites are incorporated in the References.

### Collection material and microscopic examination

Histological preparations of exo-erythrocytic stages were obtained from the collections of International Reference Centre for Avian Haematozoa (Queensland Museum, Queensland, Australia), Natural History Museum (London, UK), Nature Research Centre (Vilnius, Lithuania) and the US National Parasite Collection (National Museum of Natural History, Washington DC, USA). All accessed preparations were studied. An Olympus BX61 light microscope (Olympus, Tokyo, Japan) equipped with an Olympus DP70 digital camera and imaging software AnalySIS FIVE (Olympus Soft Imaging Solution GmbH, Münster, Germany) was used to examine preparations and prepare illustrations.

## Results

### Exo-erythrocytic development of avian malaria parasites

Information about exo-erythrocytic development of avian malaria parasites of the genus *Plasmodium* comes mainly from studies carried out by human malaria researchers who used these bird parasites, mainly of subgenera *Haemamoeba* and *Giovannolaia* as models for better understanding of human disease between the 1930s and 60s (reviews in [2–5, 7–9, 52, 54, 55]. Raffaele [56] provided first data about exo-erythrocytic development in birds experimentally infected with *Plasmodium elongatum*. The discovery and excellent illustrations of exo-erythrocytic meronts of *Plasmodium gallinaceum* in brain capillaries and other tissues of domestic chickens by James and Tate [57, 58] was the first real evidence of tissue stages in malaria parasites. These studies markedly stimulated the development of avian malariology and also activated the search of tissue stages in *Plasmodium* parasites of mammals. Exo-erythrocytic development of several *Haemamoeba* and *Giovannolaia* malaria parasites were subjects of experimental research in many laboratories in America and Europe (Table 1). However, after the discovery of malaria parasites in rodents and monkeys, which are closer to malaria parasites of humans in many biological and genetic characteristics and also are more convenient model organisms for the laboratory experimental research, the interest of human malariologists in avian malaria parasites gradually decreased in the 1960s [3, 4, 9, 53]. Because of the difficulties in designing experiments with parasites of wild birds and the unidentified vectors of many *Plasmodium* species, the exo-erythrocytic development of the great majority of avian malaria parasites of subgenera *Giovannolaia, Novyella* and *Huffia* remains unknown or only fragmentary information about their secondary exo-erythrocytic merogony is available (Table 1).

**Table 1:** Exoerythrocytic stages reported in different avian *Plasmodium* parasites

Subgenus and species	Stage			Reference
	Cryptozoite	Metacryptozoite	Phanerozoite	
*Haemamoeba*				
* Plasmodium cathemerium*	+	+	+	[54, 59, 60]
* P. gallinaceum*	+	+	+	[7, 8, 10, 54, 55, 57, 58, 61–63]
* P. giovannolai*	−^a^	−	+	[64, 65]
* P. subpraecox*	−	−	+	[66]
* P. lutzi*	−	−	+	[67]
* P. matutinum*	−	–	+	[2, 68–72]
* P. relictum*	+	+	+	[8, 54, 73–76]
* P. tejerai*	−	−	+	[60, 77–79]
*Giovannolaia*				
* P. circumflexum*	−	−	+	[80–84]
* P. durae*	−	−	+	[53, 85–89]
* P. fallax*	+	+	+	[55, 90, 91]
* P. gabaldoni*			+	[92]
* P. garnhami*	+	+	+	[2, 93]
* P. homocircumflexum*	−	−	+	[94]
* P. lophurae*	+	+	+	[2, 55, 95–97]
* P. octamerium*	−	−	+	[98]
* P. pinottii*	−	−	+	[2, 99]
* P. polare*	−	+	−	[2, 100–103]
*Novyella*				
* P. bertii*	−	−	−	[104]
* P. dissanaikei*	−	−	+	[105]
* P. hexamerium*	−	−	+	[106]
* P. nucleophilum*	−	−	+	[60, 107, 108]
* P. paranucleophilum*	−	−	+	[109]
* P. vaughani*	−	+	+	[2, 110, 111]
*Bennettinia*				
* P. juxtanucleare*	−	−	+	[2, 112–114]
*Huffia*				
* P. elongatum*	−	−	+	[2, 55, 115–118]
* P. hermani*	−	−	+	[119–122]
* P. huffi*	−	−	+	[2, 123, 124]

Only parasites, which species identification was supported by morphological or molecular identifications were included in this table. Reports of unidentified parasites or the parasites of undetermined or questionable taxonomic status were not included. To date, approximately 50 avian *Plasmodium* species were described, and 738 lineages of these parasites were reported (according to MalAvi database, http://mbio-serv2.mbioekol.lu.se/Malavi)

^a^Exoerythrocytic stages were not seen

The complete exo-erythrocytic development is known only for three species of subgenus *Haemamoeba*, and three species of subgenus *Giovannolaia* (Table 1). The tissue stages and the sequence of their development have been relatively well studied in *Plasmodium cathemerium, Plasmodium fallax, P. gallinaceum*, *Plasmodium garnhami, Plasmodium lophurae*, and *Plasmodium relictum.* Experiments with these parasites were relatively easy to design because their vectors (mosquitoes of the genera *Culex, Aedes* and some others) have been identified and colonized. Additionally, susceptible avian hosts (chickens, ducklings, turkeys, domestic pigeons, and canaries) of these parasites are easy to maintain in the laboratory [2, 4, 5, 52–55, 58, 61, 73, 74, 93, 125]. In these parasites, the general pattern of exo-erythrocytic development is similar, and it is likely to be similar in other species of these subgenera. Mainly, sporozoites induce exo-erythrocytic development in the cells of mesodermal origin, particularly often in the cells of lymphoid-macrophage system and the endothelial cells lining capillaries. Erythrocytic meronts and gametocytes develop in red blood cells. The exo-erythrocytic merogony can be arbitrarily classified in two main stages: the primary (pre-erythrocytic) merogony, which occurs before parasitaemia, and the secondary (post-erythrocytic) merogony, which develops during parasitaemia and maintains at the latency stage. The primary exo-erythrocytic merogony consists at least of two generations of meronts, which are named the cryptozoites and metacryptozoites, respectively. The secondary exo-erythrocytic merogony includes several generations of meronts named the phanerozoites. It is important to note that the development of phanerozoites can be induced by merozoites developed in the erythrocytic meronts, which is not the case in malaria parasites of mammals [2, 4, 5, 11].

**Fig. 1:**
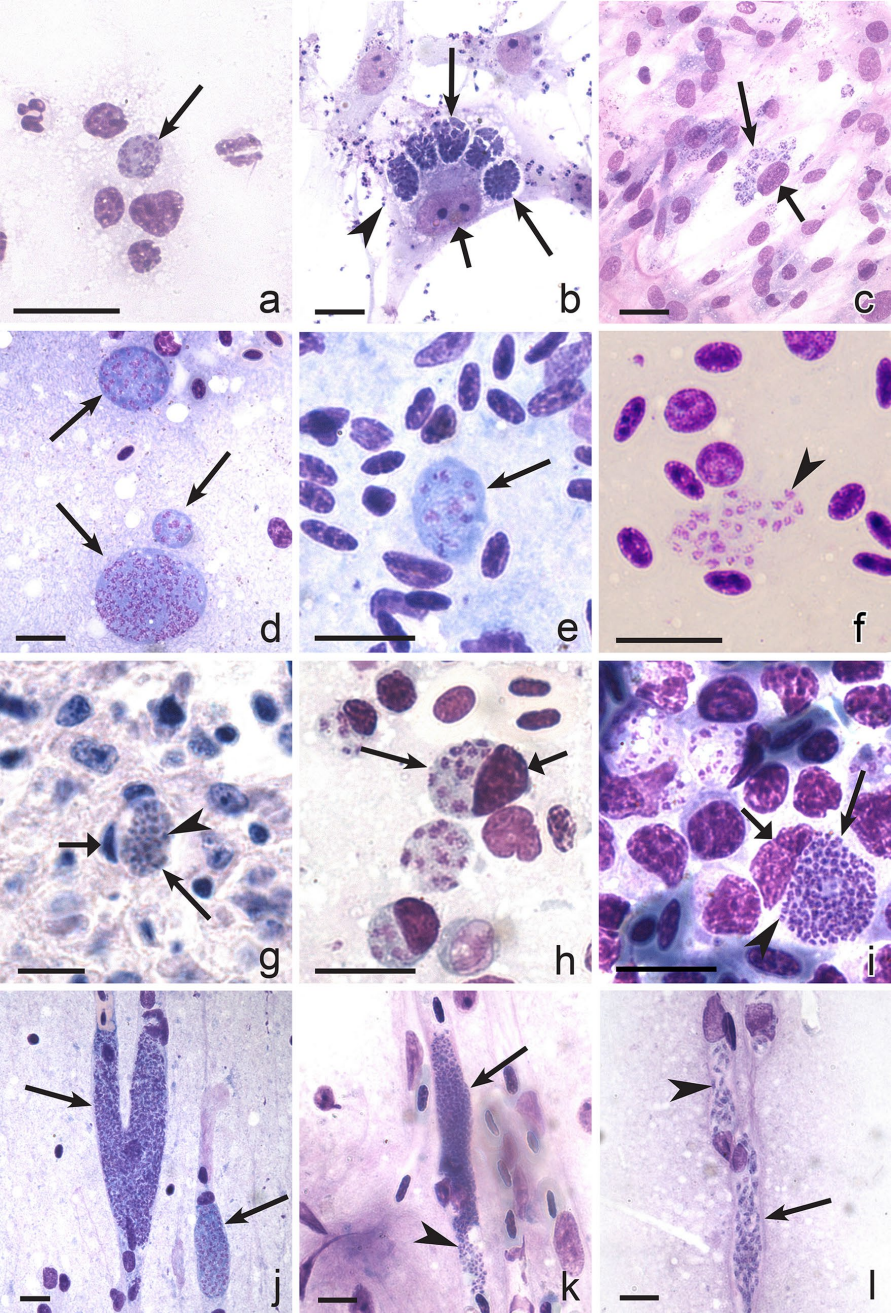
Exo-erythrocytic stages of avian malaria parasites: cryptozoites (**a**–**c**), metacryptozoites (**d**) and phanerozoites (**e**–**l**). *Plasmodium cathemerium* in wing skin of a domestic canary *Serinus canaria* approximately 2.5 days after inoculation of sporozoites (note small size of maturing cryptozoite, **a**). *Plasmodium fallax* in culture of brain cells from a turkey *Meleagris gallopavo* embryo (note five mature cryptozoic meronts and numerous mature dispersed merozoites, **b**). *Plasmodium lophurae* in culture of brain cells from a turkey embryo (note numerous small mature cryptozoic meronts located in groups, **c**). *Plasmodium garnhami* in histological section of liver of the hoopoe *Upupa epops* experimentally infected by sporozoites (note the plentiful basophilic cytoplasm and prominent different size nuclei in three metacryptozoic meronts in different stages of growth, **d**). Growing phanerozoic meront of *Plasmodium gabaldoni* in the spleen of the pigeon *Columba livia* infected by inoculation of infected blood (note the plentiful basophilic cytoplasm and prominent nuclei, **e**). Mature merozoites of *Plasmodium gabaldoni* in a brain smear of turkey experimentally infected by inoculation of infected blood (note prominent irregular shape nuclei in mature merozoites, **f**). Mature phanerozoic meront of *Plasmodium durae* in spleen of turkey experimentally infected by inoculation of infected blood (note small size of the mature meront and a deformed host cell nucleus, **g**). *Plasmodium elongatum* in a smear of bone marrow of a European greenfinch *Carduelis chloris* infected by inoculation of infected blood (note three intracellular parasites in different stages of growth in stem cells and one extracellular meront, **h**). *Plasmodium huffi* in a smear of bone marrow of a toco toucan *Ramphastos toco* experimentally infected by inoculation of infected blood (note one mature meront and several growing meronts, which markedly displace and deform host cell nuclei, **i**). Elongate phanerozoic meronts of *Plasmodium gallinaceum* blocking cerebral capillaries (note two meronts in different stages of growth, **j**). Mature phanerozoic meronts of *Plasmodium pinottii* in a brain smear of experimentally infected domestic pigeon *Columba livia* (**k**, **l**): note numerous roundish merozoites (micromerozoites, **k**) and elongate merozoites (macromerozoites, **l**) in cerebral capillaries. *Simple long arrows* meronts, *triangle wide shot arrows* host cell nuclei, *simple arrowheads* merozoites. *Scale bars* 10 μm

Mosquitoes inject sporozoites in birds and initiate development of the first generation of primary exo-erythrocytic meronts (cryptozoites), which develop in the reticular cells of many organs and tissues, including the skin (Fig. 1a–c) [54, 55, 73, 91]. The localization of cryptozoites in bird body depends on the mode of infection. After inoculation of sporozoites into skin, the cryptozoites often develop initially in macrophages or fibroblasts (Fig. 1a), sometimes in lymphocytes and fat cells, located close to the site of the mosquito bite [2, 4, 54, 95]. If sporozoites are injected intravenously, the cryptozoites mainly develop in lymphoid-macrophage cells in various internal organs; they are particularly often seen in the Kupffer cells of the liver and in the lymphoid-macrophage cells of spleen, bone marrow and other organs [5, 10, 53, 55, 73, 91, 97]. Cryptozoites (Fig. 1a–c) look like thin-walled, small roundish bodies, which usually do not exceed 20 μm in their largest diameter and produce small number of merozoites (most often fewer than 50). Cryptozoites mature rapidly (approximately between two and three days post-infection). A residual body remains after maturation of cryptozoites of some malaria parasite species [2, 4, 61]. The merozoites developing in cryptozoites are roundish, oval or slightly elongate bodies possessing prominent nuclei (Fig. 1b); they cannot infect the blood cells, so there is no parasitaemia after maturation of the cryptozoites. These merozoites induce the second generation of primary exo-erythrocytic meronts (metacryptozoites), which develop in reticuloendothelial cells, particularly in macrophages, and can be found all over the body; they are often reported in spleen, liver and lungs [2, 7, 8, 54, 55, 61, 69, 74, 93, 126]. Metacryptozoites are covered by a thin wall; they look similar to cryptozoites, but are larger and produce a greater number of merozoites (Fig. 1d). Maturation of metacryptozoites is usually asynchronous, and the first parasites mature approximately four to five days post-infection. The merozoites from metacryptozoites are morphologically similar to merozoites from cryptozoites. Metacryptozoites produce merozoites which can: (1) induce further generations of metacryptozoites; (2) initiate development of phanerozoites; and, (3) penetrate the red blood cells and give rise to agamic stages (trophozoites and erythrocytic meronts) and gametocytes. At this stage of malaria, parasitaemia develops and starts to increase, but is often still light. The time from the inoculation of sporozoites into birds until the maturation of the first generation of metacryptozoites is equal to a prepatent period.

Development of the secondary exo-erythrocytic stages (phanerozoites) can be induced in three different ways [10, 54, 55, 61, 64, 65, 67, 69, 73, 82, 91, 92, 94, 97, 117, 127]. First, a proportion of the merozoites from the erythrocytic meronts penetrate the endothelial cells of capillaries and cells of the lymphoid-macrophage system in many organs, including the brain, but particularly often in the spleen and lungs, and produce phanerozoites (Fig. 1e–l). The development of phanerozoites of *Plasmodium* species can be easily initiated in susceptible birds by merozoites from the erythrocytic meronts, and this feature is often used in experimental research when birds are exposed by the inoculation of infected blood [59, 82, 92, 94, 117, 127]. Second, a proportion of the merozoites developing in the metacryptozoites induce development of phanerozoites. Third, merozoites from phanerozoites can induce further generations of phanerozoites. It is important to note that the secondary exo-erythrocytic merogony (post-erythrocytic merogony or phanerozoite development) is absent in the malaria parasites of mammals, in which only sporozoites initiate exo-erythrocytic development whether directly, or after a delay, when they are responsible for relapses [11].

Maturation of the first generation of phanerozoites often leads to marked increase of parasitaemia. The first phanerozoites usually mature between 10 days and 3 weeks after infection with sporozoites [2, 4, 5, 55, 61, 69, 80, 91]. Phanerozoites develop asynchronously, resulting in the presence of parasites of different maturity in organs of infected birds (Fig. 1h–j). The morphology of phanerozoites is similar to that of metacryptozoites, but the former usually are larger and contain a greater number of merozoites. Over 100 merozoites usually develop in phanerozoites [4].

Both phanerozoites and erythrocytic meronts produce merozoites, which are responsible for maintenance of parasitaemia during the chronic stage of the infection. In addition, phanerozoites are responsible for relapses [2, 55, 69, 70, 91]. Because the merozoites from erythrocytic meronts, metacryptozoites and phanerozoites can initiate secondary exo-erythrocytic merogony, the course of secondary exo-erythrocytic development is complicated and difficult to follow stage by stage. The precise number of generations of phanerozoites is unknown, and it probably varies markedly in different species of parasites and avian hosts.

Both the localization and the sequence of appearance of cryptozoites, metacryptozoites and phanerozoites in different organs and tissues varies markedly depending on mode of infection, parasite species, parasite strain characteristics, age of birds, intensity of infection, stage of infection, and some other factors [4, 10, 53–55, 61, 73, 91, 92, 114, 115, 128].

Exo-erythrocytic meronts (cryptozoites, metacryptozoites, phanerozoites) are covered by a thin wall (Fig. 1a–l). These parasites most often look like roundish or oval bodies containing basophilic cytoplasm and variable numbers of nuclei, which markedly decrease in size as parasites mature (Fig. 1a–e, g–i). The shape of phanerozoites developing in the endothelial cells of capillaries and sinusoids might be determined by the shape of these structures, so they may be elongate, branching or even lobular in shape (Fig. 1j–l); branching and lobular forms particularly occur in brain (Fig. 1j) and muscles. Cytomeres do not usually develop during maturation of exo-erythrocytic meronts of avian *Plasmodium* parasites.

It is difficult to calculate the number of merozoites in mature exo-erythrocytic meronts because several parasites can develop in one cell or adjacent cells (Fig. 1c), and the boundaries between the parasites are often hardly visible, particularly when parasites are in capillaries (Fig. 1j). The number is very variable however, and fewer than 1000 merozoites usually develop in the exo-erythrocytic meronts of the majority of species of avian malaria parasites. Even young meronts displace the nuclei of infected cells and they can enucleate the host cells, however, they do not usually cause enlargement of nuclei of the host cells (Fig. 1c, g), which is a characteristic feature of exo-erythrocytic development of avian malaria parasites.

Two markedly different types of merozoites have been described in phanerozoites: micro- and macromerozoites (Fig. 1k, l). These have been reported in *P. fallax, Plasmodium giovannolai, Plasmodium pinotti* and some other species [2, 4, 55, 65, 91, 99]. The micromerozoites predominate in all avian malaria infections; these are small roundish or slightly oval bodies (approximately 1 μm in diameter), each possessing a prominent nucleus and a portion of cytoplasm (Fig. 1f, i, k). Macromerozoites are elongate (between 2 and 5 μm in length), with slightly curved bodies, and one end often slightly rounded; each merozoite possesses a prominent nucleus, and a tiny vacuole was visible in some parasites (Fig. 1l). The role of macromerozoites in the life cycle remains unclear.

The exo-erythrocytic development of the great majority of malaria parasites of subgenera *Huffia, Novyella* and *Bennettinia*, remains unknown, or only fragmental data are available, mainly about the development of phanerozoites. The primary exo-erythrocytic meronts (cryptozoites and metacryptozoites) remain undescribed in species belonging to these subgenera because of insufficient experimental research on infections via mosquito bites. Phanerozoites have been reported in some species of these subgenera (Table 1) mainly due to experimental infections, which were induced by inoculation of infected blood.

The exo-erythrocytic merogony of species of subgenus *Huffia* occurs mainly in cells of the haemopoietic system [55, 56, 116, 117, 120, 123, 124]. Phanerozoites (Fig. 1h, i) are especially numerous in migrating cells of haemopoietic tissues in bone marrow and have also been reported in spleen and liver. They develop in erythroblasts, precursor cells of the erythrocytic series, sometimes in normoblasts and thrombocytes and lymphoid cells, but have also been seen in macrophages, myelocytes and some other related cells. Although some reticuloendothelial cells were seen to be parasitized (for example, macrophages), the fixed reticuloendothelial cells have never been recorded to be parasitized, and phanerozoites have not been seen in the endothelial cells of capillaries in brain or other organs in competent avian hosts, in which the *Huffia* parasites complete their life cycle and produce gametocytes. However, this pattern of development might change during development in non-competent hosts (see description below). Both the size of phanerozoites and the number of merozoites vary markedly in *Huffia* species depending on their host cells, but the phanerozoite size rarely exceeds 20 µm in diameter (Fig. 1h, i). Intracellular growing parasites often are surrounded with a light band of the cytoplasm of host cells, and they possess the basophilic cytoplasm and compact bright-stained nuclei (Fig. 1h). Phanerozoites of *Huffia* species markedly influence the nuclei of host cells (Fig. 1h, i), which is a characteristic feature of the secondary exo-erythrocytic merogony during these infections. The nuclei of infected cells are displaced, deformed and may be even pushed out from the cells (Fig. 1h, i). As a result, some phanerozoites appear to be extracellular bodies (Fig. 1h). Several phanerozoites often parasitize the same host cell during heavy infections.

It is important to note that mode of exo-erythrocytic development can change significantly when *Huffia* parasites appear in non-adapted avian hosts. For example, enormous numbers of *Plasmodium (Huffia) elongatum* phanerozoites developed in reticuloendothelial cells in the penguin *Spheniscus demersus* [129]. In this case, the parasite was found not only in cells of haemopoietic system, but also developed extensively in histiocytes of lungs, spleen, liver, heart, and some other organs. Because of changes in both morphology and site of development of the same parasite during development in different avian hosts, it might be difficult to identify species of *Plasmodium* using morphological characters of exo-erythrocytic meronts, particularly when infection occurs in unusual hosts.

Available experimental data indicate that the extensive exo-erythrocytic merogony usually lasts about a month post infection. After this period, reticuloendothelial cells can develop resistance to infection, the number of phanerozoites decreases and the parasitaemia maintains mainly due to a limited erythrocytic merogony [2, 4, 61]. It is difficult to see phanerozoites in some *Novyella* species even if parasitaemia is high [2, 4, 130–132]. Recent histological studies, combined with chromogenic in situ hybridization diagnostic tools indicate that exo-erythrocytic development in some tropical *Novyella* species is mainly primary, and the persistence of infection is due to long-lasting light parasitaemia [133]. In other words, merozoites developing in erythrocytic meronts of these parasites might be unable or have limited ability to produce phanerozoites. This method of persistence might be common in *Novyella* species, which are widespread, prevalent and diverse in tropical countries [134, 135], but more investigations are required on their exo-erythrocytic development (Table 1). A similar mode of persistence occurs in *Plasmodium malariae* in humans [11].

**Fig. 2:**
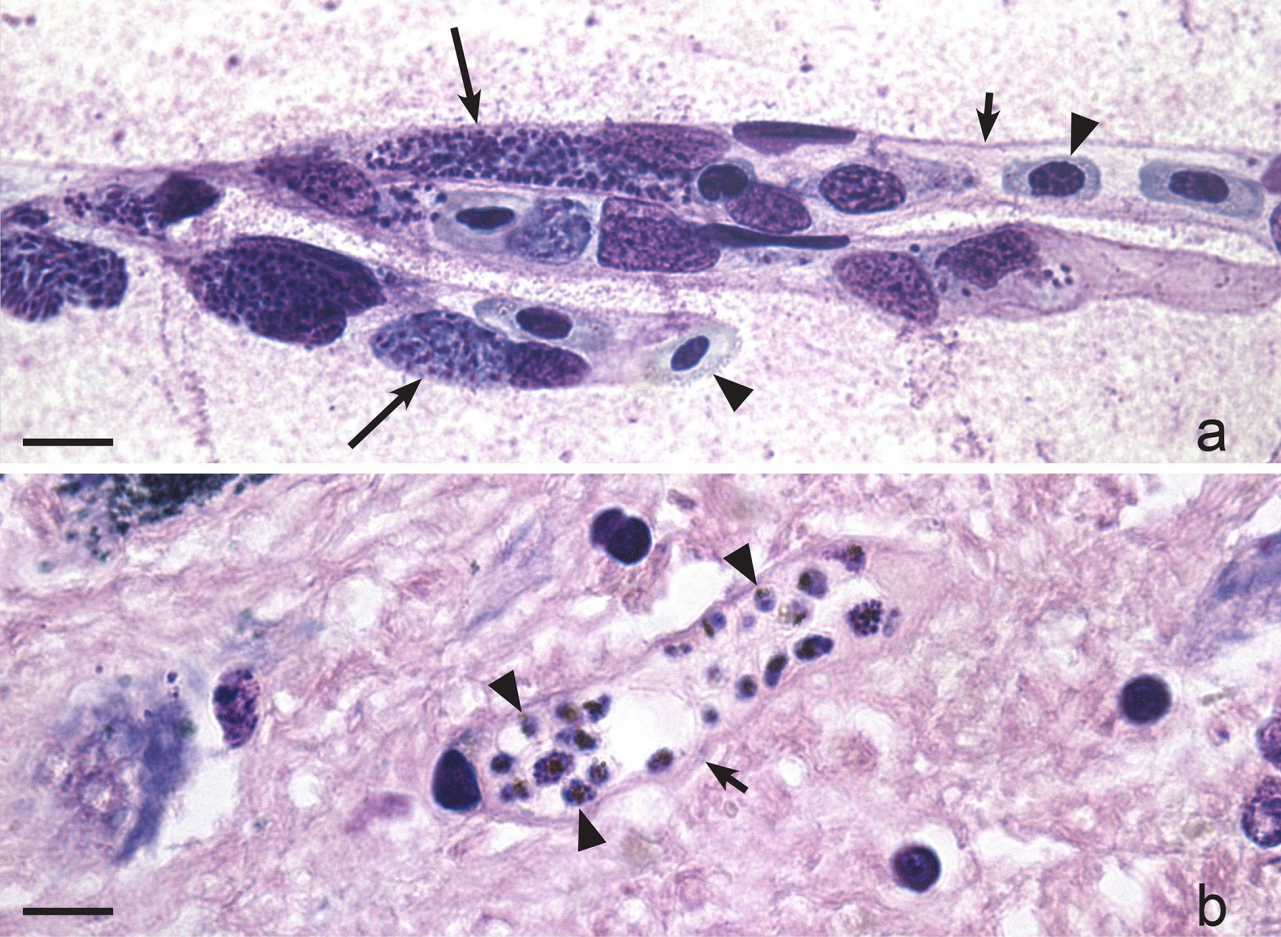
Cerebral malaria in a chicken (**a**) and a man (**b**) due to *Plasmodium gallinaceum* (**a**) and *Plasmodium falciparum* (**b**) infections, respectively. Note numerous developing and mature phanerozoites in endothelial cells of brain capillaries (**a**) and numerous infected red blood cells, which adhere to the endothelial cells of a brain capillary (**b**). In both malaria cases, the circulation interrupts due to blockage of the capillaries by the parasites, and the ischaemic brain changes occur, resulting in severe diseases and similar clinical symptoms, which mechanisms are different. *Simple long arrows* phanerozoites, *simple short arrows* wall of capillaries, *triangle arrowheads* red blood cells. *Scale bars* 10 μm

Brain damage leading to ischaemic changes is among the most severe pathologies caused by the human malaria parasite *Plasmodium falciparum* and many species of avian malaria, but the mechanisms of pathology during human falciparum malaria and bird malaria are different [4, 10, 12, 53, 94, 136, 137]. Both in birds and humans, cerebral malaria is due to interruption of the circulation in brain vessels (Fig. 2a, b). However, birds are dying because of development of phanerozoites in endothelial cells of the capillaries: the large parasites follow the shape of the vessels, which are eventually completely blocked by the parasites (Fig. 2a). During *P. falciparum* infection, the infected red blood cells adhere to the endothelial cells of capillaries of the brain (Fig. 2b), leading to interruption of the circulation and resulting in ischaemic brain changes, with similar clinical symptoms and health consequences as is a case of malaria in birds.

### Exo-erythrocytic development of avian garniids

Species of Garniidae are diverse and parasitize many species of reptiles in countries with warm climates. These parasites are similar to species of the Plasmodiidae in that merogony occurs in the cells of fixed tissues and also blood cells of hosts, but malarial pigment (haemozoin) is absent from all stages of their development [138, 139]. Garniids are absent from temperate and cold climates probably due to lack of specific vectors, which remain unidentified [6, 139, 140]. The development of garniids in their vertebrate hosts is similar to that of *Plasmodium* species in birds, and some species described as garniids belong to this genus [141]. Molecular characterization is needed to specify taxonomic position of haemosporidian species currently belonging to the Garniidae.

Only one species of the Garniidae, *Fallisia neotropicalis* is known in birds [4, 142]. In this parasite primary exo-erythrocytic meronts, developing from sporozoites have not been reported. Secondary exo-erythrocytic merogony (phanerozoites) can be induced by merozoites developing in the cells of the peripheral blood, as is the case in many species of avian malaria parasites. The phanerozoites look similar to the phanerozoites described in bird malaria parasites. Gabaldon et al. [142] reported that they develop in the reticular cells and in the histiocytes of the connective tissues in many organs including the brain.

It is worth mentioning that there is no DNA sequence information on parasites of the majority of genera and subgenera of the Garniidae, so there is inadequate genetic data to assess their phylogenetic relationships with other haemosporidians. In other words, the entire family is missing from phylogenetic analyses of haemosporidians [15, 17, 143]. That is a shortcoming of the currently available studies on evolution of haemosporidian parasites from the point of view of the limited taxon sampling.

### Exo-erythrocytic development of avian haemoproteids

Approximately 150 species of avian haemoproteids (Haemoproteidae) belonging to the genus *Haemoproteus* (subgenera *Haemoproteus* and *Parahaemoproteus*) have been described [4, 18, 144], and recent molecular studies indicate that many more species probably exist [17]. However, there is, at best, only fragmentary information on exo-erythrocytic development in the great majority of the species (Table 2). Exo-erythrocytic meronts were discovered by Aragão [161] in pigeons naturally infected with *Haemoproteus columbae* in Brazil. The large parasites with cytomeres were seen in endothelial cells of lungs. It was concluded that merozoites from these meronts penetrated in the red blood cells and produced gametocytes. That was the first evidence of tissue stages in haemosporidian parasites. In spite of the long history of research, the general patterns of the exo-erythrocytic development of avian *Haemoproteus* species remains insufficiently understood mainly because of the paucity of experimental studies addressing this issue [5].

**Table 2:** Exoerythrocytic stages reported in different avian *Haemoproteus* parasites

Subgenus and species	Stage		Reference
	Meront	Megalomeront	
*Parahaemoproteus*			
* Haemoproteus attenuatus*	+	−^a^	[66 ^b^, 145]
* H. balearicae*	+	–	[146]
*H. coatneyi*	+	–	[147]
* H. halcionis*	–	+	[148]
* H. handai*	+	+	[149]
* H. lophortyx*		+	[20]
* H. mansoni*	+	+	[150, 151]^c^
* H. minutus*		+	[23, 26]
* H. nettionis*	+	–	[152]
* H. orizivorae*	+	–	[153]
* H. passeris*	+	+	[154–157]
* H. picae*	+	–	[158]^d^
* H. sacharovi*	–	+	[5, 31]
*Haemoproteus*			
* H. columbae*	+	+	[41, 132, 148, 157, 159–164]
* H. palumbis*	+	–	[165]

Only parasites, which species identification was supported by morphological or molecular identifications were included in this table. Reports of unidentified parasites or the parasites of undetermined or questionable taxonomic status were not included. To date, approximately 150 avian *Haemoproteus* species were described, and 945 lineages of these parasites were reported (according to MalAvi database, http://mbio-serv2.mbioekol.lu.se/Malavi)

^a^Exoerythrocytic stages were not seen

^b^Garnham, Duggan [66] attributed this parasite to *Haemoproteus danilewskii*

^c^Originally identified as *H. meleagridis*, which possibly is a synonym of *H. mansoni* (see [4])

^d^Garvin et al. [158] attributed this parasite to *Haemoproteus danilewskii*

**Fig. 3:**
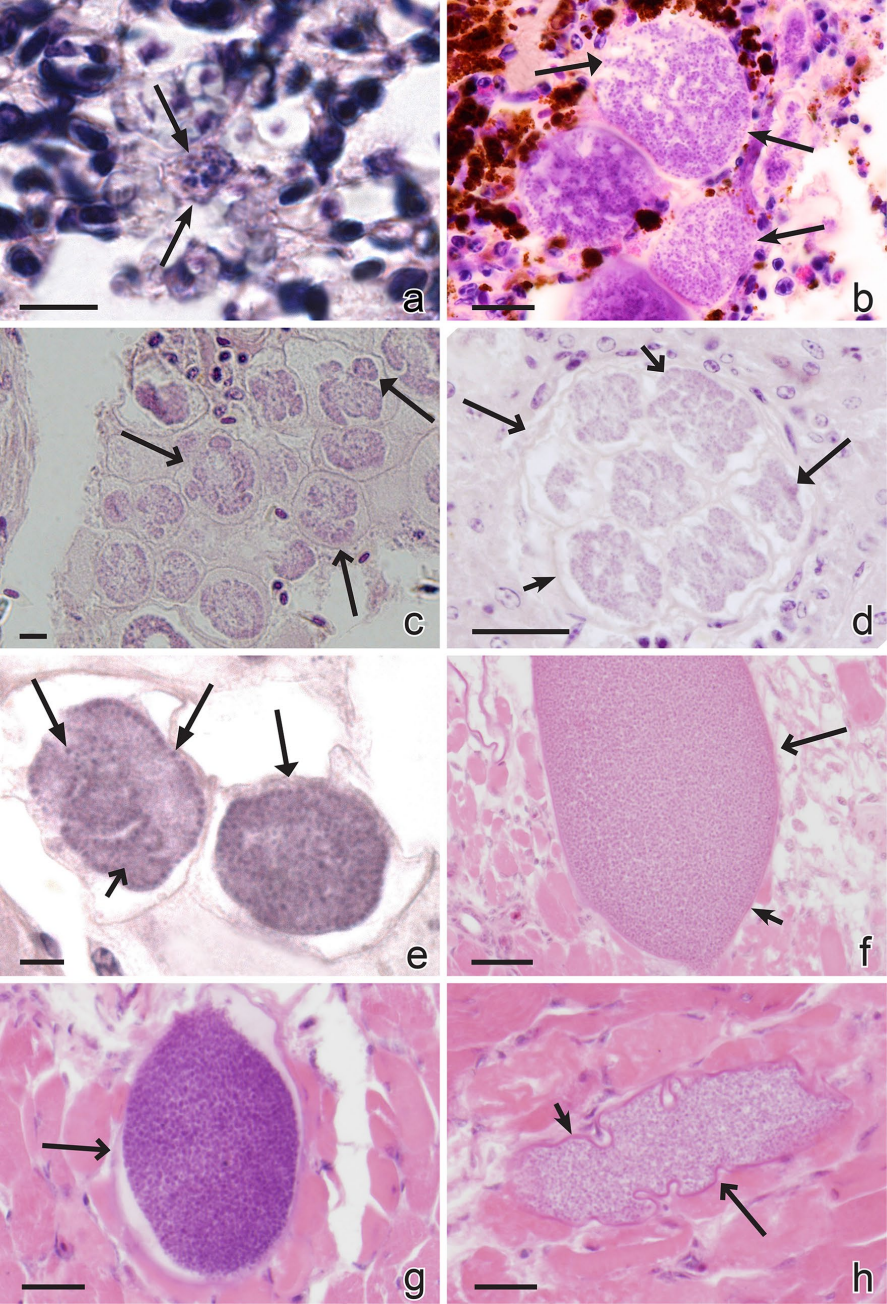
Exo-erythrocytic stages of *Haemoproteus* parasites: meronts (**a**, **b**) and megalomeronts (**c**–**h**). Small mature meront of *Haemoproteus palumbis* in lungs of a naturally infected nestling of the English wood-pigeon *Columba palumbis* (**a**). Group of maturing meronts of *Haemoproteus attenuatus* in lungs of a naturally infected European robin *Erythacus rubecula* (note numerous irregular-shaped cytomeres with developing merozoites, **b**). Numerous developing megalomeronts of *Haemoproteus passeris* in a section of liver in a naturally infected House sparrow *Passer domesticus* (note that megalomeronts tend to group, and each megalomeront is covered by a wall and contains several cytomeres, **c**). A maturing megalomeront of *H. passeris* (the same preparation as in **c**; note that the parasite is covered by a thick wall and contains seven well-defined cytomeres, which are covered by a thin membrane and contain numerous irregularly shaped subcytomeres with developing merozoites, **d**). Two maturing cytomeres of *H. passeris* (the same preparation as in **c**; note that the cytomeres separate from each other before maturation, and nuclei locate on the edge of subcytomeres, **e**). Mature oval megalomeront of *Haemoproteus mansoni* (syn. *Haemoproteus meleagridis*) in the section of the pectoral muscle of a turkey *Meleagris gallopavo* (note that megalomeront is surrounded by thick wall and is overfilled with roundish merozoites; the muscle fibres surrounding the parasite are swollen and pale-stained, **f**). Rupturing mature roundish megalomeront of *H. mansoni* (the same preparation as in **f**; note the swollen tissue surrounding the parasite, **g**). Mature lobular-shape megalomeront of *H. mansoni* (the same preparation as in **f**; note irregular outline of the parasite, **h**). *Simple long arrows* meronts, *simple wide long arrows* megalomeronts, *triangle long arrows* parasite nuclei, *triangle wide long arrows* cytomeres, *simple wide short arrows* subcytomeres, *simple short arrows* megalomeront wall. *Scale bars* 10 μm (**a**,** b**,** e**), 50 μm (**c**,** d**,** f**–**h**)

Haemoproteids do not multiply in blood cells, and only tissue merogony occurs in birds [2, 4, 5, 166]. Two types of the exo-erythrocytic meronts develop (Table 2; Fig. 3a–h): (1) the thick-walled megalomeronts, which usually exceed 100 μm and sometimes might reach up to 1000 μm in their largest diameter (Fig. 3c–h); and, (2) the thin-walled meronts (Fig. 3a, b), which are smaller (usually less than 50 µm). Morphology of the both types of meronts is markedly variable, and roundish, oval, elongate, branching, and lobular-shaped parasites have been described. Growing megalomeronts and large meronts are usually split into individual portions (cytomeres) (Fig. 3b–e), in which multiplication is continued, and the parasite nuclei are often seen along the edge of developing cytomeres (Fig. 3e). The division of large parasites into cytomeres and the peripheral arrangement of nuclei facilitate metabolic functions. It is important to note that nuclei of host cells are not enlarged either by meronts or megalomeronts of haemoproteids. From this point of view, *Haemoproteus* parasites are similar to avian *Plasmodium* species, but are different from *Leucocytozoon* spp.

Meronts (Fig. 3a, b) and megalomeronts (Fig. 3c–h) are found in various organs, with no clear preference for particular organs or tissues in most well-examined *Haemoproteus* and *Parahaemoproteus* species (Table 3). However, there are exceptions. For example, the exo-erythrocytic merogony of *Haemoproteus mansoni* (syn. *Haemoproteus meleagridis*), *Haemoproteus handai* and *Haemoproteus lophortyx* is often seen in skeletal muscle tissues (Fig. 3f–h), and this preference is confirmed by experimental infections [20, 149–151]. Limited available data indicate some patterns of exo-erythrocytic development in certain haemoproteid species, but they might be different in different parasites. That needs additional investigation because much information comes from single or few naturally infected birds, with unclear origin or fluctuations of the infections through time [31, 41, 43, 145, 148, 155, 157, 159, 164, 167, 168].

**Table 3:** Location of exoerythrocytic stages in different avian haemoproteids

Location of tissue meronts	Parasite subgenus and species	
	*Haemoproteus*	*Parahaemoproteus*
Lungs	*H. columbae, H. palumbis*	*H. attenuatus, H. balearicae, H. coatneyi, H. nettionis, H. orizivorae, H. passeris, H. picae, H. sacharovi*
Spleen	*H. columbae*	*H. attenuatus, H. coatneyi, H. lophortyx, H. mansoni, H. nettionis, H. picae*
Heart	*H. columbae, H. palumbis*	*H. coatneyi, H. handai, H. mansoni, H. minutus, H. nettionis*
Liver	*H. columbae*	*H. coatneyi, H. passeris, H. picae*
Skeletal muscles (particularly often in pectoral muscle)	*H. columbae*	*H. halcionis, H. handai, H. mansoni, H. lophortyx*
Kidneys	*H. columbae*	*H. coatneyi, H. passeris*
Proventiculus	*H. columbae*	*H. handai*
Gizzard	*H. columbae*	*H. sacharovi*
Caecum	–^a^	*H. coatneyi*
Tongue	–	*H. handai*
Hip	–	*H. handai*

References for a given parasite species are the same as in Fig. 2. Only parasites, which species identification was supported by morphological or molecular identifications were included in this table. Reports of unidentified parasites or the parasites of undetermined or questionable taxonomic status were not included

^a^Exoerythrocytic stages were not seen

Megalomeronts (Fig. 3c–h) were described in several species of *Parahaemoproteus* and *Haemoproteus* (Table 2). These parasites seriously damage affected organs and tissues (Fig. 3e, f), particularly after their maturation and rupture. Megalomeronts of various parasite species have been seen in the skeletal muscle, heart, gizzard, lungs, liver, spleen, kidney, and proventriculus of their hosts [20, 21, 31, 41, 149, 154, 155, 157, 168]. Atkinson et al. [150, 151] reported that skeletal muscles were particularly often parasitized during experimental *H. mansoni* infection in domestic turkeys (Fig. 3f–h) however, the megalomeronts were also recorded in cardiac muscle. Interestingly, the *H. mansoni* megalomeronts were not seen in the liver, lungs, brain, kidney, bone marrow, femur, gizzard, duodenum, pancreas, or cecum. Most of these organs, especially lungs and brain, are highly vascular. This suggests a specific requirement for myofibroblasts or their associated physiological environment for the development of megalomeronts. Miltgen et al. [149] also found megalomeronts predominantly in muscle tissues, in the heart, tongue, hip and pectoral muscle; these were especially numerous in the pectoral muscle. Megalomeronts of *H. minutus* developed in myocardial and skeletal muscles and some other organs of captive parakeets, and the infection was lethal in these avian hosts [23, 26].

Due to their size, the largest megalomeronts can sometimes be seen without a microscope, on the surface of damaged organs [5, 31]. Large parasites are surrounded by a thick hyaline wall (Fig. 3d, f, h). Cytomeres are well defined in developing megalomeronts (Fig. 3d), and nuclei are typically arranged on the periphery of developing cytomeres (Fig. 3i). Megalomeronts are often seen located close to each other, forming large groups (Fig. 3c).

The number of generations of exo-erythrocytic meronts before development of parasitaemia has only been sufficiently documented in *H. mansoni*, in which at least two generations of the meronts develop in the skeletal and cardiac muscles before the parasite produce merozoites that are able to invade red blood cells [150, 151]. First-generation meronts were seen in the endothelium of capillaries and in myofibroblasts of experimentally infected domestic turkeys. They matured approximately 5 days after infection with sporozoites. The mature parasites can reach up to 20 μm in largest diameter and contain elongate merozoites, which are 5–6 μm in length. The elongate merozoites induce the next generation of merogony in the endothelial cells of the capillaries and in myofibroblasts and also initiate development of meronts in the reticular cells of the spleen. The meronts of the second generation are megalomeronts, which are covered by a hyaline wall (Fig. 3f, h). Megalomeronts mature approximately 17 days after infection and produce numerous roundish merozoites with a diameter of about 1 μm. The merozoites formed in megalomeronts penetrate into red blood cells and become gametocytes.

The overall number of generations of meronts after single-sporozoite infections is unknown in avian haemoproteids, but there are certainly many generations because infected birds usually maintain parasitaemia for many years in controlled laboratory conditions. Additionally, the spring relapses, which are induced by dormant tissue meronts were reported in avian haemoproteids [2, 4, 5]. Available data indicate that meronts of *H. mansoni,* causing relapses and maintaining chronic parasitaemia in turkeys, develop in reticular cells of the spleen [151].

It is important to note that avian haemoproteids can develop tissue stages in ‘wrong’ avian hosts, in which sporozoites initiate exo-erythrocytic development, which is then arrested (aborted), so merozoites and gametocytes do not appear. Such abortive infections might be virulent and even lethal in non-adapted avian hosts, but remain insufficiently investigated. Recent PCR-based studies [21–23] have supplemented earlier histopatology research [41, 151] and provided first real evidence that species of *Haemoproteus* and *Parahaemoproteus* are responsible for disease and even mortality in birds. It was shown that common species and lineages of *Haemoproteus* parasites might be relatively benign in naturally adapted hosts, but cause lethal disease in non-adapted birds due to damage of organs by megalomeronts [21–23, 26, 41]. The tissue stages of *Haemoproteus* species in dead hosts resemble the megalomeronts of *Leucocytozoon* spp.; it was difficult to determine the disease etiology solely based on morphological data [33–35, 38, 39, 42–44, 169–172]. The traditional opinion about the harmlessness and insignificant veterinary importance of avian haemoproteids [44] requires partial reconsideration [21, 22, 26, 30]. The true extent of pathology and mortality caused by *Haemoproteus* parasites requires additional investigation, particularly in wildlife because death of infected birds has been reported before the development of parasitaemia. Such disease is difficult to diagnose using blood samples alone, whether either by microscopy or PCR-based tools. Experimental studies are needed, but remain rare because of their difficult design, which requires laboratory manipulations of wild birds and obtaining live sporozoites. In haemoproteids, the latter can mainly be collected by experimental infection of biting midges and hippoboscid flies.

Application of a chromogenic in situ hybridization (ISH) method for detection of tissue meronts in bird organs might be helpful during investigation of exo-erythrocytic merogony of haemosporidians, as is the case in avian *Plasmodium* parasites [22, 45, 94]. This method speeds up the search for tissue stages of malaria parasites, but has not been used in diagnosis of leucocytozoids and has been rarely applied in avian haemoproteid research [22]. It is important to note that the traditional histology techniques remain informative in haemosporidian parasite morphology research and are useful for better understanding pathologies caused by haemoproteid infections in different organs and tissues (Fig. 3). It worth using these methods in parallel during investigation of exo-erythrocytic development of haemosporidian parasites [94].

### Exo-erythrocytic development of avian leucocytozoids

Approximately 40 species of avian leucocytozoids have been described. These parasites do not multiply in blood cells, and only tissue merogony occurs, as is the case in *Haemoproteus* parasites [2, 4, 166, 173]. The exo-erythrocytic meronts have been found and described in 14 leucocytozoid species (Table 4), and merogony was studied particularly well in several species of subgenus *Leucocytozoon* (*Leucocytozoon danilewskyi, Leucocytozoon fringillinarum, Leucocytozoon dubreuili, Leucocytozoon simondi, Leucocytozoon smithi)* and one species of *Akiba* (*Leucocytozoon caulleryi*) using sporozoite-induced infections [162, 177, 179, 195, 197, 199, 202, 206, 209, 211, 217, 221, 226]. The exo-erythrocytic development occurs in the parenchymal cells of the liver (hepatocytes), in tubular cells of kidneys, in macrophages and various other reticuloendothelial cells, including endothelial cells of the capillaries.

**Table 4:** Exoerythrocytic stages reported in different avian *Leucocytozoon* parasites

Subgenus and species	Stage		Reference
	Meront	Megalomeront	
*Leucocytozoon*			
* Leucocytozoon artamidis*	+	+	[174]
* L. berestneffi*	+	−^a^	[4, 175, 176]
* L. danilewskyi*	+	+	[177]
* L. dubreuili*	+	−	[178–180]
* L. fringillinarum*	+	−	[179]
* L. lovati*	+	+	[181–183]
* L. macleani*	+	−	[184, 185]
* L. marchouxi*	−	+	[186]
* L. podargii*	−	+	[187]
* L. sakharoffi*	+	+	[176, 188–192]
* L. simondi*	+	+	[162, 166, 193–209]
* L. smithi*	+	−	[210–213]
* L. tawaki*	+	−	[214, 215]
*Akiba*			
* L. caulleryi*	+	+	[216–227]

Only parasites, which species identification was supported by morphological or molecular identifications were included in this table. Reports of unidentified parasites or the parasites of undetermined or questionable taxonomic status were not included. To date, approximately 40 *Leucocytozoon* species were described, and 694 lineages of these parasites were reported (according to MalAvi database, http://mbio-serv2.mbioekol.lu.se/Malavi)

^a^Exoerythrocytic stages were not seen

**Fig. 4:**
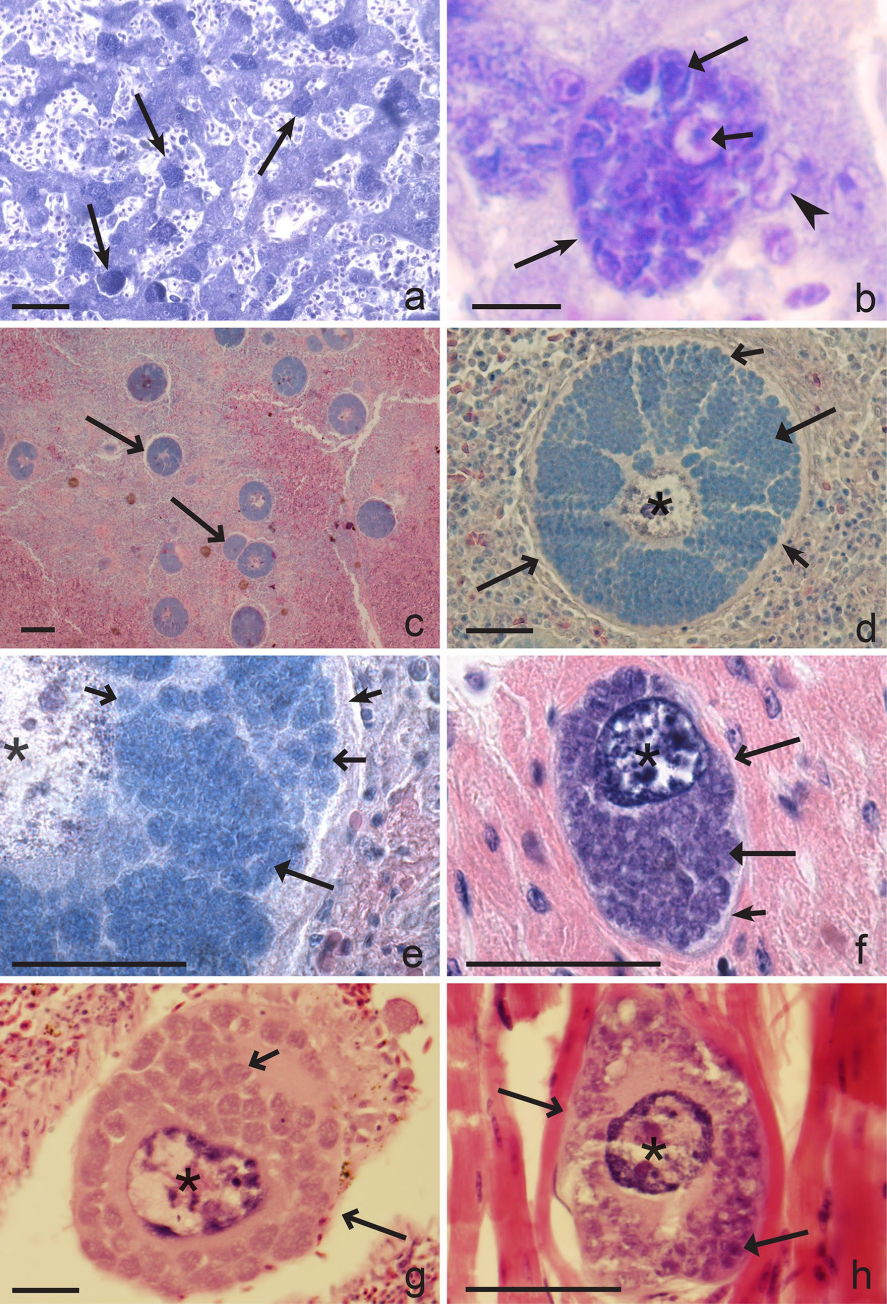
Exo-erythrocytic stages of *Leucocytozoon* parasites: meronts (**a**, **b**) and megalomeronts (**c**–**h**). Hepatic meronts of *Leucocytozoon simondi* in a liver section of an experimentally infected Pekin duck *Anas platyrhynchos domestica* (note numerous meronts and the much haemorrhagic and disorganized liver tissue, **a**). Maturing hepatic meront of *L. simondi* (the same preparation as in **a**; note numerous cytomeres, in which division of parasite nuclei occurs and non-changed size of the infected hepatocyte nucleus, in comparison to nuclei size in the adjacent non-infected hepatocytes, **b**). Megalomeronts of *Leucocytozoon sakharoffi* in different stages of growth in spleen of a naturally infected the hooded crow *Corvus cornix* (note numerous roundish megalomeronts in different stages of growth, **c**). Maturing megalomeront of *L. sakharoffi* (the same preparation as in **c**; note a markedly enlarged host cell nucleus or the ‘central body’ of megalomeront, capsular-like wall surrounding the parasite, and numerous cytomeres, **d**). A fragment of the *L. sakharoffi* megalomeront (the same preparation as in **c**; note cytomeres containing numerous roundish subcytomeres, in which nuclear division occurs, a well-evident ‘central body’ and capsular-like wall surrounding the megalomeront, **e**). Megalomeront of *L. simondi* in hart of a naturally infected mallard *Anas platyrhynchos* (note numerous cytomeres, a well-evident ‘central body’, and capsular-like wall, **f**). Megalomeront of *Leucocytozoon* sp. in a section of spleen in a naturally infected Australian magpie *Cracticus tibicen* (note a large ‘central body’ and numerous roundish cytomeres in a parasite developing in a vessel, **g**). Megalomeront of *Leucocytozoon* sp. in a section of skeletal muscle of a naturally infected tawny frogmouth *Podargus strigoides* (note a large ‘central body’ and numerous roundish developing cytomeres; the muscle fibres surrounding the parasite are swollen, **h**). *Simple long arrows* meronts, *simple wide long arrows* megalomeronts, *triangle wide long arrows* cytomeres, *simple wide short* subcytomeres, *simple short arrows* megalomeront wall, *triangle wide short arrow* a nucleus of infected hepatocyte, *simple arrowhead* a nucleus of non-infected hepatocyte, *stars* host cell nucleus or ‘central body’ of megalomeront. *Scale bars* 10 μm (**b**), 50 μm (**a**,** d**–**h**), 200 μm (**c**)

Several generations of tissue meronts develop in all experimentally examined *Leucocytozoon* species but the precise number of generations remains unclear [4, 173, 176, 179, 190, 197–199, 206, 211, 226]. The sporozoites initiate exo-erythrocytic development in the parenchymal cells of the liver, in which the hepatic meronts of the first generation grow (Fig. 4a, b). The primary hepatic merogony inside hepatocytes is a general pattern in the exo-erythrocytic development of species of the subgenus *Leucocytozoon*. From this point of view, species of this subgenus are more similar to malaria parasites of mammals than to avian *Plasmodium, Haemoproteus* or *Akiba* parasites [4, 11]. In *L. smithi*, exo-erythrocytic merogony takes place only in parenchymal cells of the liver [201, 211, 213]. However, in other *Leucocytozoon* species various organs and tissues can be affected, particularly by subsequent (induced by merozoites) generations of tissue meronts. In *L. simondi*, the first generation of tissue meronts develop only in hepatocytes (Fig. 4a, b), and only subsequent generations of meronts appear in other organs and cells. In *L. dubreuili* and *L. fringillinarum*, sporozoites initiate development of meronts in liver, but also in kidneys where they locate in renal tubular cells [177–180]. As meronts grow, nuclei of the host cells increase in size and the cytoplasm breaks down into numerous cytomeres, in which nuclear fission continues (Fig. 4b). The development of first generation meronts is completed four to five days post infection. Numerous uninuclear merozoites develop in each cytomere. It is important to note that the hepatic meronts usually do not induce marked enlargement of nuclei in hepatocytes (Fig. 4b). Mature meronts are very variable in size, but are usually smaller than 50 μm in diameter; they produce merozoites, which are roundish bodies of approximately 1–2 μm in diameter. Merozoites invade various blood cells producing the gametocytes. Some merozoites also initiate new generations of meronts in liver and the kidneys. The hepatic and renal merogonies are not synchronized, and young growing meronts may be seen together with the mature parasites.

During *L. dubreuili* infection, some of the sporozoites invade liver parenchymal cells and produce small (about 6 μm in largest diameter) oocyst-like meronts, which produced elongate (cystozoite-like) merozoites germinating from a single germinal centre [178]. These meronts are probably persistent (dormant) stages, which are responsible for relapses; they have not been reported in other *Leucocytozoon* parasites.

After rupture of the hepatic meronts, the cytoplasmic ‘fragments’ develop in parallel with merozoites. These fragments are called the syncytia (islands); they are covered by a plasma membrane and contain several nuclei [166, 173, 198]. Syncytia are remnants of the meronts. They are unable to penetrate into host cells, but are washed out into the circulation, spread over the bird body and are phagocytized by macrophages and other cells of the reticuloendothelial system giving rise to large meronts—megalomeronts (megaloschizonts) (Fig. 4c–h). The term megalomeront (megaloschizont) was suggested by Huff [162] for the ‘host cell-*Leucocytozoon* parasite’ complex. The markedly enlarged nucleus of the host-cell (‘central body’ of megalomeront) is the most readily distinguishable character of megalomeronts in *Leucocytozoon* species (Fig. 4c–h). The central body is often located close to the centre of megalomeronts (Fig. 4d, g, h). Syncytia grow rapidly and induce marked enlargement both of the nuclei and the cytoplasm of host cells. Numerous cytomeres appear (Fig. 4d–f). As the parasite matures, each cytomere breaks up into numerous smaller bladder-like portions (subcytomeres, Fig. 4d, e, g), with the nuclei often located on the surface of the subcytomeres. Subdivision of smaller cytomeres continues until uninuclear merozoites appear. Both the division of parasite into cytomeres and the peripheral arrangement of nuclei in the cytomeres facilitate the metabolic and trophic functions. Nuclei of host cells, which contain mature megalomeront, might reach 50–100 μm in diameter and even greater (Fig. 4d, g). The greatly enlarged nucleus of the host cell (the ‘central body’ of megalomeront) probably takes part in transportation of nutrient materials inside the parasites. Growth of megalomeronts from their initiation to maturity represents a several 1000-fold increase in volume. Mature megalomeronts are packed with numerous merozoites, and boundaries between the cytomeres gradually disappear.

Megalomeronts are surrounded with a thick capsule-like wall of fibrillar structure, which is collagen-positive [166, 173, 195, 198]. The wall can be about 1 µm in width or even thicker. The encapsulation is most obvious in organs with a dense reticular network such as the spleen and lymph nodes (Fig. 4d, e), and it is least in the brain and heart (Fig. 4f). Growing megalomeronts displace surrounding cells and reticular fibres, which become associated with the surface of the expanding sphere and thus take part in the formation of the capsular wall.

Mature megalomeronts are very variable in size, which usually ranges between 50 and 500 µm, but might be even greater. The largest megalomeronts can be seen with the naked eye on the surface of heavily infected organs [4, 166, 173, 195]. It is likely that size of mature megalomeronts partially depends to the size and number of syncytia, which are phagocytized by host cells [166, 195, 199, 202]. Development of megalomeronts is asynchronous due to asynchronous phagocytosis of syncytia in different organs (Fig. 4c).

Megalomeronts have been reported all over body in the infected birds, including brain, nerves, heart, pericardium, lungs, trachea, kidneys, liver, pancreas, intestine, skeletal muscles, masticatory, and glandular parts of stomach, gonads, bursa of Fabricius, but most often seen in the spleen (Fig. 4c–e) and the lymph nodes [162, 166, 173, 186, 191, 192, 195, 205]. Intensity of infection in the latter two organs can be extremely high (Fig. 4c), and the parasites can represent over 50% of the infected spleen mass. The marked enlargement of spleen is sometimes due to the megalomeronts mass per se during intense *Leucocytozoon* infection [195], and such mechanism of spleen enlargement has not been recorded in any other haemosporidiosis. It should be noted that megalomeronts are usually uncommon in the liver, which is the site of extensive phagocytosis in birds. It seems that the absence or rarity of megalomeronts in the liver is due to local immunity induced after intense primary hepatic merogony (Fig. 4a), and the destruction of syncytia in the Kupffer cells.

Megalomeronts develop rapidly and become mature approximately four to five days after ingestion of syncytia by their host cells or approximately 7–9 days following a sporozoite infection [166, 195, 200, 202]. Many thousands of uninuclear merozoites develop in each megalomeront. The merozoites are approximately of 1 μm in diameter; they inhabit various blood cells and produce gametocytes.

Some of the merozoites formed in megalomeronts are phagocytized by the reticuloendothelial cells and slow down their development, periodically producing megalomeronts, which are responsible for the chronic parasitaemia and also spring relapses [208, 209]. Persisting meronts are few and usually difficult to find. They were seen in lungs during *L. simondi* infection [199].

Small meronts develop in all species of leucocytozoids, but megalomeronts do not. The latter were reported in *L. danilewskyi, Leucocytozoon sakharoffi, L. simondi*, *L. caulleryi* and in some other species (Table 4). Megalomeronts were not found in *L. dubreuili, L. fringillinarum, L. smithi* and many other species. The role of megalomeronts in the life cycle of leucocytozoids is insufficiently understood. In *L. simondi* and *L. danilewskyi*, the merozoites from megalomeronts inhabit blood cells and initiate the development of gametocytes, which markedly change the host cells by producing elongate host cell processes [177, 228]. However, there is no strict correlation between the development of megalomeronts and the appearance of gametocytes in the fusiform host cells in other leucocytozoid species. For example, gametocytes do not develop in fusiform host cells in *Leucocytozoon artamidis, L. caulleryi, Leucocytozoon marchouxi, Leucocytozoon podargii* and *L. sakharoffi*, although these species do produce megalomeronts in some avian hosts [174, 186, 187, 191, 192, 226], while such gametocytes predominate in *L. smithi,* in which megalomeronts are absent [211]. Additional investigations are needed to understand the role of megalomeronts in the life cycle of leucocytozoids.

Exo-erythrocytic merogony of *L. (Akiba) caulleryi* differs from the species of subgenus *Leucocytozoon* in several characters [216, 218, 221, 224, 226]. First, the meronts of the first and following generations do not develop in hepatic cells. This feature is more similar to species of *Plasmodium* and *Haemoproteus* than to species of *Leucocytozoon*. All generations of meronts of *L. caulleryi* grow in the endothelial cells of the capillaries of many organs and can be found in brain, bursa of Fabricius, thymus, trachea, bronchus, lungs, heart, liver, spleen, kidneys, pancreas, gullet, masticatory and glandular stomach, crop, duodenum, ovary, testis, oviduct, and various skeletal muscles. It is interesting to note that they occur not only in the visceral organs but also in the eyes and sciatic nerves [219]. The meronts of *L. caulleryi* were especially numerous and often seen in bursa of Fabricius and thymus.

Second, merozoites developing in meronts of the first generation are elongate (up to 7 μm in length) and are similar morphologically to the first generation merozoites described in *H. mansoni* [5, 223, 226].

**Fig. 5:**
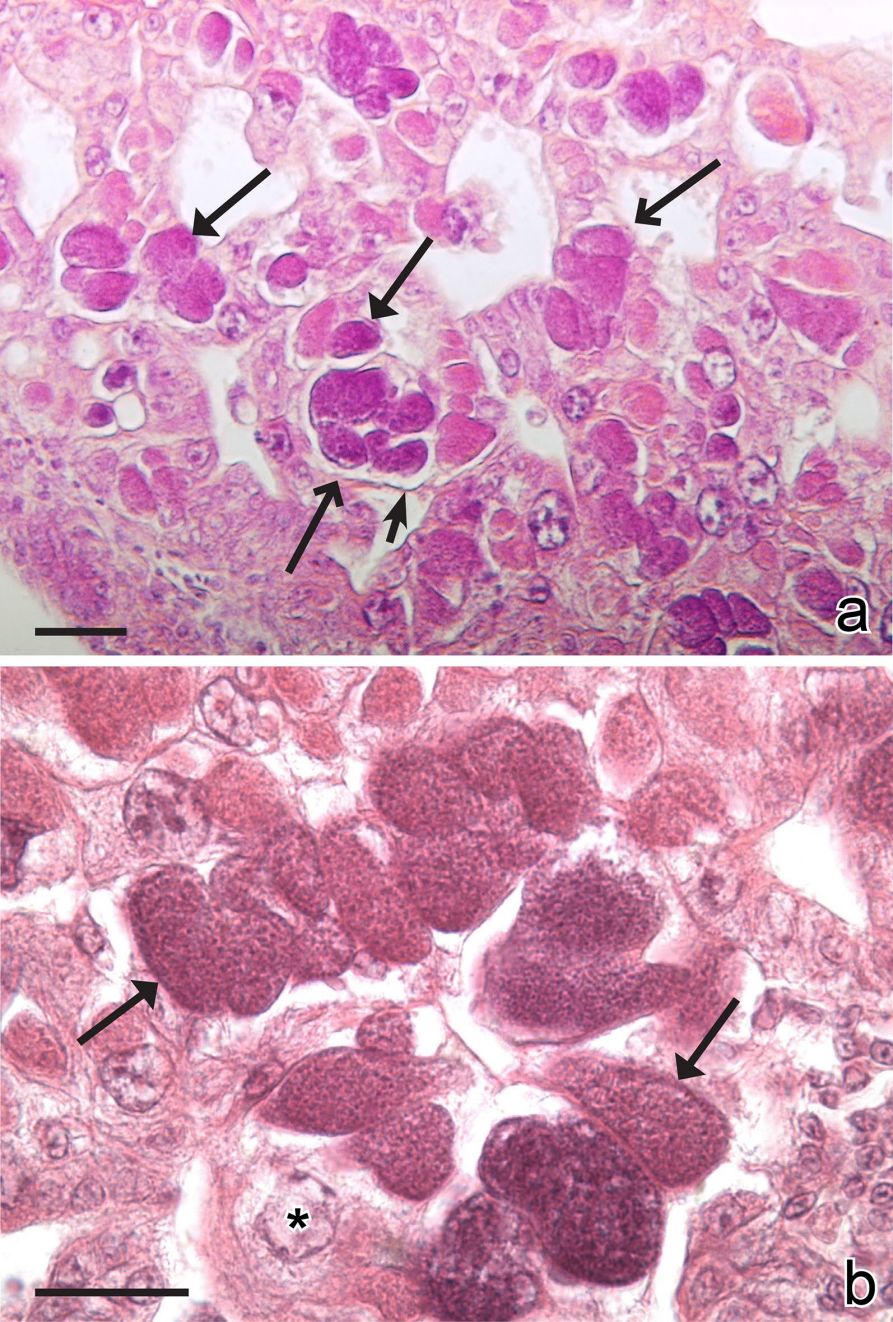
Developing megalomeronts of *Leucocytozoon (Akiba) caulleryi* in a kidney section of an experimentally infected domestic chicken *Gallus gallus domesticus* (**a**, **b**). Note numerous maturing extracellular cytomeres; developing megalomeronts are enclosed within a well-defined envelope (**a**). Host-cell nuclei are slightly enlarged and, if seen, locate on edge of the cytomere masses (**b**). *Simple wide long arrows* megalomeronts, *triangle wide long arrows* cytomeres, *simple short arrow* megalomeront envelope, *star* host cell nucleus of megalomeront. *Scale bars* 50 μm

Third, megalomeronts complete their development extracellularly and they do not have the ‘central body’ [221, 223, 224, 226, 228]. Extracellular development occurs when the infected cells rupture, and growing megalomeronts containing numerous cytomeres are released from the cells (Fig. 5a, b). Extracellular developing megalomeronts (Fig. 5b) are seen in many organs and tissues. Megalomeronts may be solitary or in groups (Fig. 5a), and they complete maturation extracellularly, a unique character in described haemosporidian parasites. Large megalomeronts can reach more than 300 μm in diameter [221, 222]. The size of megalomeronts depends on their location. Solitary megalomeronts are usually larger than the parasites developing in clusters [220]. Megalomeronts are enclosed within a well-defined envelope (Fig. 5a). The host-cell nucleus is enlarged and, if present in growing megalomeronts, is located on the edge of the cytomeres, but not within the cytomere masses, as is the case in megalomeronts of *Leucocytozoon* species (compare Figs. 4d–h, 5b).

## Discussion

In spite of limited knowledge about the exo-erythrocytic development in many species of haemosporidian parasites, some basic morphological characters of the tissue meronts indicate their generic or subgeneric identity. These characters are given in Table 5 and can be helpful for determining the taxonomic status of tissue stages in histological preparations of organs and tissues, particularly in samples collected from naturally infected birds.

**Table 5:** Main morphological characters of exoerythrocytic meronts of avian haemosporidian parasites

Character	*Plasmodium*	*Haemoproteus*	*Leucocytozoon*	*Akiba*
Merogony in hepatocytes (Fig. 4a, b)	Absent	Absent	Present	Absent
Merogony in cells of hemopoietic system (Fig. 1h, i)	Present	Absent	Absent	Absent
Thin-walled elongate meronts in brain capillaries (Fig. 1j–l)	Present	Absent	Absent	Absent
Megalomeronts (Fig. 3c–h, Fig. 4c–h, Fig. 5a, b)	Absent	Present	Present	Present
‘Central body’ in megalomeronts (Fig. 4d–h)	Absent	Absent	Present	Absent
Extracellular development of megalomeronts (Fig. 5a, b)	Absent	Absent	Absent	Present
Prominent (more than threefold in comparison to controls) enlargement of host cell nuclei (Fig. 4d, f–h)	Absent	Absent	Present	Absent

Absence of a given character in examined parasite preparations might be due to inappropriate stage of parasite development or other methodology issues, and should be treated with caution in practical work. Presence of a given character in preparations indicates taxonomic status of haemosporidians

During mammalian malaria, exo-erythrocytic development occurs in hepatic cells

This review shows that the exo-erythrocytic development of the majority of species of wildlife haemosporidian parasites remains unknown or studied only fragmentally (Tables 1, 2 and 4), most often using incidentally collected samples from wild, naturally infected birds, in which the fate and duration of these infections is unclear. Molecular diagnostic tools have opened new opportunities to detect infections and to determine parasite species identity in tissue stages, providing the first real evidence that these avian parasites are more virulent than formerly believed, causing severe damage in various organs of non-adapted avian hosts [21, 22, 26, 29, 30, 94].

It is important to note that the exo-erythrocytic merogony of a given strain of haemosporidian parasites may differ significantly in different avian hosts, sometimes resulting in abortive development and increase of virulence of parasites in ‘wrong’ hosts. This phenomenon is well documented, but its mechanisms remain insufficiently understood. For example, extensive exo-erythrocytic development of *P. elongatum* in non-adapted penguins occurs not only in cells of haemopoietic system, as usually is the case in adapted passerines, but also in reticuloendothelial cells, resulting in the intense merogony, the marked damage of various organs and severe disease, during which parasitaemia might be absent [4, 129]. Similar abortive exo-erythrocytic development occurs in *H. minutus* infection of captive parakeets, in which sporozoites produce megalomeronts that cause lethal disease, but parasitaemia is absent as well [23, 26]. The lethal aberrant avian haemosporidioses caused by tissue stages have been reported in various bird species, using of PCR-based diagnostic methods in Europe, America and Australia [20–26]. Available molecular data leave no doubt that aberrant haemosporidian infections may kill non-adapted avian hosts. However, it is unclear how often that happens in wildlife and what are the consequences of such infections for bird populations.

Many haemosporidian species and their lineages have broad specificity and can infect birds belonging to different species, genera, families, and even orders [4, 5, 13, 14, 16, 19]. The exo-erythrocytic development of a given species of avian haemosporidian can be different in different avian hosts. In other words, the sequence of different stages and their occurrence during the exo-erythrocytic development of a particular parasite strain are not stable characteristics, but they can be functions of the host species. For example, experimental studies showed marked differences in the exo-erythrocytic development of the same strain of *Leucocytozoon* in different vertebrate hosts [200, 201]. Mainly, a strain of *L. simondi* developed megalomeronts in ducks, but not in geese. Complete exo-erythrocytic development occurred only ducks. In both species of avian hosts, the infective for vectors stages (gametocytes) developed. The ‘partial’ exo-erythrocytic development in geese was accompanied by reduced parasitaemia and virulence of this parasite strain. Moreover, the parasite was finally eliminated from infected geese, resulting in lack of relapses, which was not the case in ducks, in which long-lasting parasitaemia and relapses occurred. It seems that ducks are less adapted to some strains of *L. simondi,* resulting in development of megalomeronts and heavy disease. During development in geese, the same strain undergoes only the ‘partial’ development, and megalomeronts do not occur. This finding was supported experimentally by exposure of ducks and geese to natural infection of *L. simondi* at different localities in the upper peninsula of Michigan [200]. Interestingly, *L. sakharoffi* is cosmopolitan and infects many species of the Corvidae, but megalomeronts were reported only in the hooded crow *Corvus cornix* [4, 192]. Additionally, *Leucocytozoon marchouxi* is common in doves and pigeons, but its megalomeronts were reported only in the Mauritian pink pigeon *Columba mayeri* [186]. It seems that these parasites also develop megalomeronts only in non-adapted avian hosts. Thus, the available data show that the exo-erythrocytic development of haemosporidian parasites is relatively flexible, and it can be modified when the same parasite strain appears in different species of avian host. However, it remains unclear how often such changes occur in wildlife. This issue is related to severity of disease and bird health, and it needs additional investigation. It is essential to expand the application of chromogenic in situ hybridization in research of haemosporidians from simply diagnostic purposes [22, 45, 47, 94] towards species-level detection of localization of the exo-erythrocytic stages and pathology associated with these stages in various tissues. Sampling of naturally dead birds in zoos, aviaries and wildlife as well as experimental infections of captive birds are most straightforward ways to for such research [20–25, 29, 94].

## Conclusion

The exo-erythrocytic development of avian haemosporidian parasites requires much additional research. Importantly, the tissue merogony of some avian haemosporidian species and their strains is not as stable as was formerly believed, and it might differ significantly in different avian host species. Understanding the mechanisms of this phenomenon is an important question for future research. Additionally, the use of molecular diagnostic tools and histological methods in parallel have allowed recognition of the existence of underestimated manifestation of haemosporidian infections, which cause diseases and even mortality in birds due to damage of organs by tissue stages. In the latter case, the parasites initiate the exo-erythrocytic development, but cannot complete it in ‘wrong’ hosts, resulting in the abortive merogony. A problem of the abortive haemosporidian development is related to health both in bird and blood-sucking insects, but remains largely unexplored [22, 27, 30, 229]. This study calls for research on the exo-erythrocytic development, which might broadly inform strategies to mitigate wildlife haemosporidioses due to identifying patterns of occurrence, pathology and mechanisms of abortion in still neglected agents of avian infection. A methodology combining the traditional histological techniques with molecular diagnostic tools is essential to speed research in this field of avian malariology. The prominent knowledge gained due to research by former generations of parasitologists provides a good starting point for addressing mechanisms of exo-erythrocytic development in haemosporidians.
